# FOXO1 plays an essential role in apoptosis of retinal pericytes

**Published:** 2010-03-10

**Authors:** Mani Alikhani, Sayon Roy, Dana T. Graves

**Affiliations:** 1 Department of Orthodontics, New York University, College of Dentistry, New York, NY; 2 Departments of Medicine and Ophthalmology, Boston University School of Medicine, Boston, MA; 3 Department of Periodontics, University of Medicine and Dentistry of New Jersey, Newark, NJ

## Abstract

**Purpose:**

An early and significant event in diabetic retinopathy is the loss of retinal microvascular pericytes. Studies were performed to investigate pathways through which an advanced glycation endproduct and tumor necrosis factor (TNF)-α stimulate apoptosis in retinal pericytes through the activation of the pro-apoptotic transcription factor Forkhead box O1 (FOXO1).

**Methods:**

Human retinal pericytes were stimulated by carboxymethyllysine (CML)-collagen, an advanced glycation endproduct, or TNF-α in vitro. Apoptosis was assessed by measuring cytoplasmic histone-associated DNA. The role of FOXO1 was examined by RNA interference (RNAi), and specific inhibitors were used to investigate the role of p38 and Jun N-terminal kinase mitogen-activated protein kinase (JNK MAP) kinases, Akt, and nuclear factor kappa B (NF-κB). Caspase-3 activity was measured with a luminescent substrate, and FOXO1 DNA-binding activity was measured by electrophoretic mobility shift assay (EMSA).

**Results:**

TNF-α and CML-collagen but not control collagen stimulated apoptosis, caspase-3 activity, and FOXO1 DNA-binding activity in pericytes. Silencing FOXO1 by small interfering RNA prevented apoptosis of pericytes in response to both TNF-α and CML-collagen. By use of specific inhibitors, we demonstrated that both FOXO1 activation and subsequent apoptosis was mediated, in part, by p38 and JNK MAP kinases. In contrast Akt and NF-κB inhibitors had the opposite effect on pericyte apoptosis.

**Conclusions:**

The results demonstrate pathways through which two different mediators, TNF-α and an advanced glycation endproduct, can induce pericyte apoptosis through activation of the transcription factor FOXO1.

## Introduction

Diabetes mellitus is the most frequent endocrine disease, causing a high degree of morbidity and contributing to elevated rates of mortality. One of the principle long-term complications of diabetes is microangiopathy, which affects various organs and contributes to diseases such as diabetic retinopathy, neuropathy, and nephropathy [1,2]. An early histopathologic feature of diabetic retinopathy is selective degeneration of pericytes in the retinal capillary vessels. It has been shown that pericytes of diabetic retinas undergo changes consistent with apoptosis [3,4]. Pericytes do not replicate in the adult retina and their degeneration contributes to increased vascular permeability and retinal edema [5,6]. The loss of pericytes is thought to result in focal retinal capillary endothelial cell proliferation, leading to microaneurysms or degeneration of endothelial cells, and forming acellular capillaries, which can lead to subsequent formation of areas of nonperfusion [7].

Mechanisms proposed to account for pericyte apoptosis include formation of advanced glycation endproducts (AGE) and retinal inflammation [8,9]. It has been shown that AGE can induce dose- and time-dependent apoptotic effects on pericytes [10]. Tumor necrosis factor (TNF)-α also has been found in human retinas with proliferative diabetic retinopathy [11,12] and has been shown to induce apoptosis of retinal endothelial cells [13]. Interestingly, anti-inflammatory drugs prevent early events in diabetic retinopathy via TNF-α suppression [14], and TNF-α inhibition in vivo reduces the loss of microvascular cells [9]. While AGE and inflammatory signals may play an important role in the process of pericyte apoptosis, it is important to consider that these events are initiating signals, and therefore it is necessary to investigate their downstream targets.

We recently demonstrated that both AGE and TNF-α can promote apoptosis by activation of the Forkhead box O1 (FOXO1) transcription factor that, in turn, changes the balance of gene expression toward apoptosis [15-17]. Interestingly, high levels of FOXO1 have been reported in diabetes, but the scope of these studies has focused on the effect of FOXO1 on mRNA levels of genes that increase glucose production, thereby contributing to hyperglycemia in diabetes [18]. Since diabetes can increase FOXO1 activity and potentiate cells toward apoptosis, it is logical to assume that FOXO1 may also play a role in apoptosis of pericytes.

The forkhead box class-O (FOXO) winged helix transcription factors are orthologs of the *Caenorhabditis elegans* forkhead factor DAF-16 [19,20]. Forkhead transcription factors FOXO1, FOXO3, and FOXO4 (formally known as FKHR, FKHR-L1, and AFX, respectively) modulate apoptosis through gene expression [19,20]. FOXO1 activation, in particular, has a global effect on apoptotic gene expression and induces approximately 25 pro-apoptotic genes that promote cell death [17]. Furthermore, FOXO1 is activated in the retina of diabetic animals and its knockdown significantly reduces formation of acellular capillaries and formation of pericyte ghosts [21].

One possible pathway through which FOXO1 can be activated in response to diabetes is through the mitogen-activated protein (MAP) kinase pathway [22]. There are three major convergence points in the MAP kinase pathway involving p38, c-Jun NH2-terminal kinase (JNK), and extracellular signal-related protein kinase (ERK). p38 and JNK in most cell types generate pro-apoptotic signals, while ERK mediates typically a survival (anti-apoptotic) signal [23,24]. The purpose of the experiments described here was to investigate whether FOXO1 plays a functional role in apoptosis of retinal pericytes induced by TNF-α and carboxymethyllysine (CML)-collagen through in vitro studies and to examine whether the MAP kinase pathway mediates FOXO1 activation, which is induced by both stimuli in microvascular pericytes.

## Methods

### Pericyte cultures

Primary bovine retinal pericytes were purchased from VEC Technologies (Rensselaer, NY). Cells were cultured in Dulbecco’s modiﬁed Eagle's medium (DMEM) medium (Cambrex, Charles City, IA) supplemented with 20% fetal bovine serum (FBS). Assays were performed at passage 2 or 3. Prior to assay cells were incubated in serum-free DMEM medium for 2 days. For all assays cells were tested at approximately 70%–80% confluence in serum-free medium. In some cases cells were preincubated for 2 h with or without signaling inhibitors purchased from Calbiochem (LaJolla, CA) as follows: p38 inhibitor SB203580 (4–4[4-fluorophenyl]-2-[4-methylsulfinylphenyl]-5-[4-pyridyl] 1H-imidazole), 10 μM [25]; the AKT inhibitor, triciribine, 1 μl [26]; and JNK inhibitory peptide H-GRKKRRQRRRPPRPKRPTTLNLFPQVPRSQDT-NH2 (L-HIV-TAT 48–57 PP-JBD20), 10 μM [27]. The concentrations of inhibitors were selected based on published studies [25-27]. The same approach was used to inhibit nuclear factor (NF)-κB with the specific NF-κB inhibitor SN50 (100 μg/ml), which was compared to incubation with SN50M control peptide that lacks activity (100 μg/ml; Biomol, Plymouth Meeting, PA). For apoptosis assays cells were incubated with TNF-α (R and D, Minneapolis, MN) or CML-collagen with or without inhibitors or small interfering (si)RNA for 24 h, as described below. For electrophoretic mobility shift assay (EMSA), cells were incubated for 1 h in assay medium with or without 20 ng/ml of TNF-α or CML-collagen (200 μg/ml). Cells were then lysed with lysis buffer (Pierce, Rockford, IL), nuclei obtained by centrifugation at 12,000x g for 10 min, and nuclear proteins isolated using nuclear extraction buffer (25 mM Tris-HCl pH 7.6, 150 mM NaCl, 1% NP-40, 1% Sodium deoxycholate and 0.1% SDS, Pierce). For caspase-3 activity lysates were prepared using cell lysis buffer provided by R and D systems. After centrifugation at 12,000x g for 10 min at 4 °C, total protein was quantified using a bicinchoninic acid (BCA) protein assay kit (Pierce). Caspase-3 activity was detected by using the specific caspace-3 fluorogenic substrate Asp-Glu-Val-Asp (DEVD) peptide conjugated to 7-amino-4-trifluoromethyl coumarin. Measurements were made on a GENios microplate reader (Tecan, Durham NC) using a filter for excitation (400 nm) and detection of emitted light (505 nm). Buffers without cell lysate and cell lysates without substrate were used as negative controls. Statistical significance was established by ANOVA (ANOVA) with Tukey’s post hoc test.

### CML-collagen

CML-collagen (AGE-collagen) was prepared by chemical modification of acid-soluble bovine skin collagen type-1 (Sigma, St Louis, MO), as previously described [28,29]. Briefly, 50 mg of collagen was dissolved in 25 ml of 1 mM HCl freshly made in sterile water and incubated at 37 °C with occasional mixing. Sterile phosphate buffered saline (PBS; PH 7.8, 25 ml) was added, followed by sodium cyanoborohydride (1.42 g) and sodium glyoxyliate acid (0.715 g). Control collagen was prepared at the same time except that no glyoxyliate acid was added. All samples were then incubated at 37 °C for 24 h. AGE collagen and control collagen were then exhaustively dialyzed against distilled water. Both CML-collagen and control collagen were soluble at the concentrations stored and tested. In total, 3%–8% of lysine residues in CML-collagen was converted to CML, as determined by the trinitrobenzenesulfonic acid assay [30]. Based on this level of modification, 200 μg/ml of CML-collagen would represent approximately 4.8 nmol/ml of carboxymethylysine, which approximates the levels of carboxymelthylysine reported in serum (approximately 2.6 nmol/ml) [31]. The collagen modification that we generated is tenfold less than the amount reported to assess CML binding and activation of NF-κB [28] and only a small amount higher than has been reported for the skin of aged or diabetic individuals [32]. Low levels of endotoxin were detected; approximately 0.1 ng/ml in both control collagen and CML-collagen samples.

### Electrophoretic mobility shift assay

Interactions between nuclear proteins and FOXO1 DNA probe (CAAAACAA) were investigated using an EMSA kit from Panomics (Redwood City, CA), following the manufacturer’s instruction and as previously reported [33]. Briefly, samples were incubated with the biotin-labeled DNA probe, followed by separation of the mixture on a non-denaturing polyacrylamide gel and assessed for shifted bands that correspond to the protein/DNA complexes. Specificity was demonstrated for each by adding 60-fold molar excess of unlabeled oligonucleotide. Each experiment was performed two to three times with similar results.

### Small interfering RNA experiments

Experiments were performed in retinal pericytes that were approximately 70%–80% confluent, as described above. Cells were transfected with siRNA (5 nM), by pre-mixing siRNA with Hiperfect transfection reagent (Qiagen) and then adding the mixture to cells following the manufacturer’s instructions. siRNAs specific for FOXO1 were designed by Qiagen to be nonhomologous with other genes. Two different FOXO1-based siRNAs were used: strongly silencing (siRNA-A, AAG CCC TGG CTC TCA CAG CAA) and partially silencing (siRNA-B, AAG TTC ATT CGT GTG CAG AAT), which correspond to sequences previously described [17]. In addition, scrambled siRNA (AAT TCT CCG AAC GTG TCA CGT) was also used as a control. Apoptosis was measured by detecting cytoplasmic histone-associated DNA (Roche, Indianapolis, IN). Experiments were performed three times. The statistical difference between samples was determined by ANOVA followed by Tukey’s multiple comparison test.

## Results

**Figure 1:**
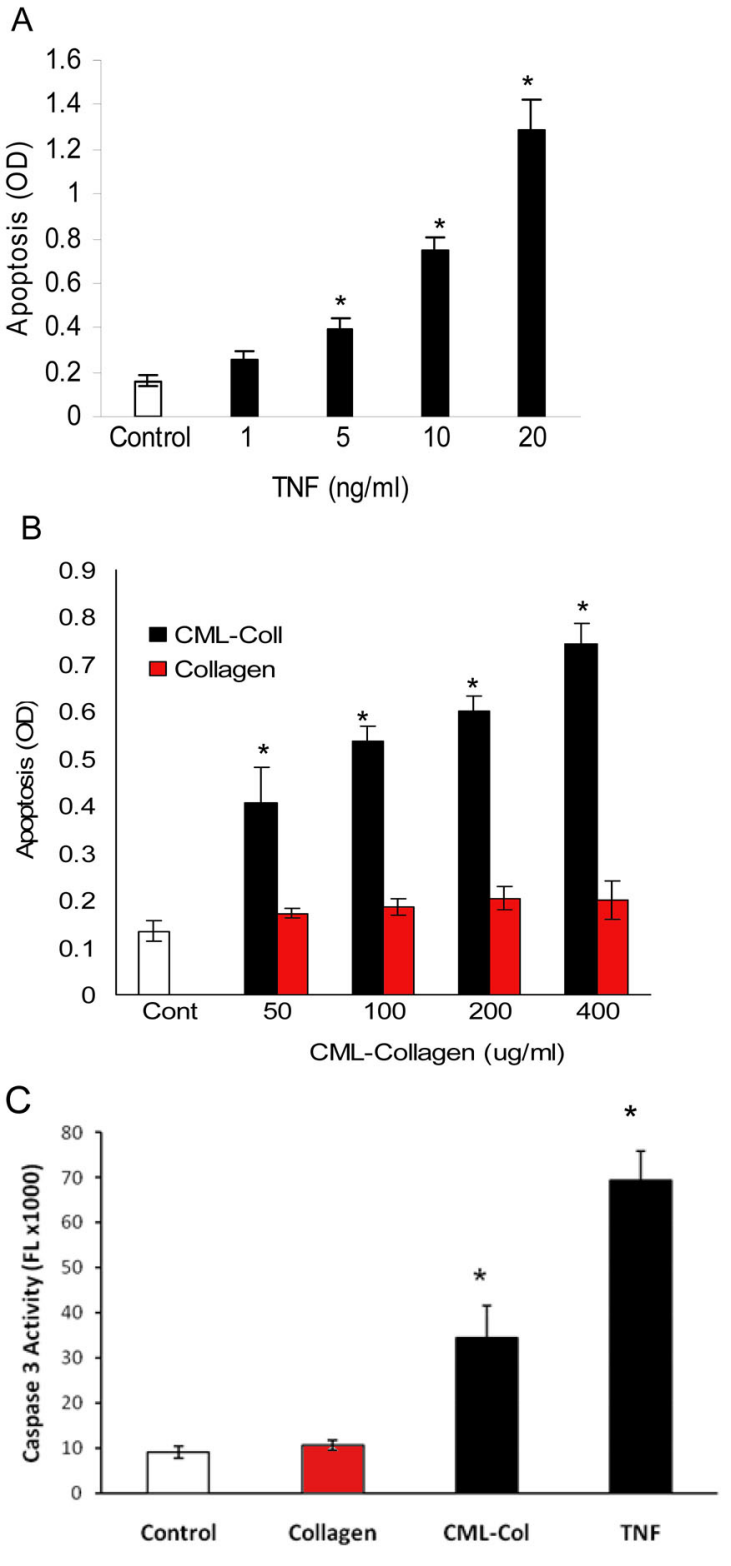
Tumor necrosis factor-alpha (TNF-α) and Advanced glycation endproduct (AGE) induce apoptosis in pericytes in vitro. Primary bovine retinal pericytes were incubated with the indicated concentration of (**A**) TNF-α (0–20 ng/ml) or (**B**) carboxymethyllysine (CML)-collagen or unmodified collagen (0–400 μg/ml) . Cells were lysed after 24 h and apoptosis was measured by ELISA measurement of histone-associated cytoplasmic DNA. **C**: Caspase-3 activity was measured by fluorometric assays in lysates from cultured primary bovine retinal pericytes incubated with CML-collagen, unmodified collagen (200 μg/ml), or TNF-α (20 ng/ml) for 24 h. Each value represents the mean of five replicates±standard error of the mean. The experiment was performed three times with similar results. *, p<0.05 significantly differs from control unstimulated cells or unmodified control collagen.

In vitro experiments demonstrated that apoptosis is increased in retinal pericytes with increased concentrations of TNF-α in a dose-dependent manner (Figure 1A). TNF-α at 20 ng/ml increased apoptosis in pericytes sevenfold compared with the control, which was statistically significant (p<0.05). Similarly, CML-collagen induced a dose-dependent increase in pericyte apoptosis, while control collagen did not (Figure 1B). CML-collagen compared with unmodified collagen induced a 2.9-fold increase in apoptosis (p<0.05; Figure 1B). When caspase-3 activity was measured, a similar increase was observed (Figure 1C). TNF-α (20 ng/ml) increased caspase-3 activity 7.8-fold compared to unstimulated cells, while CML-collagen, compared with control collagen at a concentration of 200 μg/ml, increased caspase activity 3.2-fold (p<0.05). In subsequent studies a concentration of 20 ng/ml TNF-α and 200 µg/ml CML-collagen or control collagen was used, unless stated otherwise.

**Figure 2:**
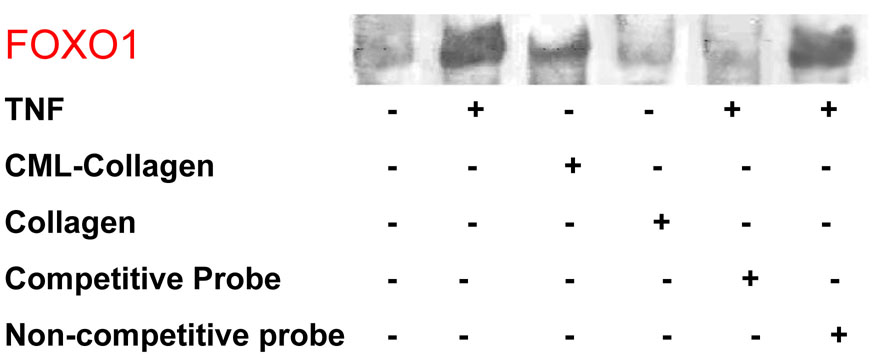
Forkhead box O1 (FOXO1) was activated in response to Tumor necrosis factor alpha (TNF-α) and advanced glycation endproduct (AGE) in retinal pericytes. Primary bovine retinal pericytes were stimulated with TNF-α (20 ng/ml), carboxymethyllysine (CML)-collagen, or collagen (200 μg/ml) for 1 h. After nuclear extraction, activation of FOXO1 was measured by electrophoretic mobility shift assay (EMSA). Unlabeled FOXO1 in excess was used as a competitive inhibitor. A probe with nonspecific sequence (nonspecific oligonucleotide) was used as a negative control. The experiment was performed three times with similar results.

Studies were undertaken to determine whether TNF-α or CML-collagen stimulated DNA-binding activity of FOXO1 by EMSA. In the absence of TNF-α or CML-collagen stimulation, insignificant amounts of FOXO1 activation were detected in retinal pericytes (Figure 2). Incubation with TNF-α (20 ng/ml) or CML-Collagen (200 μg/ml) induced FOXO1 DNA-binding activity. This was shown to be specific by competitive inhibition with excess unlabeled FOXO1 probe (Figure 2).

**Figure 3:**
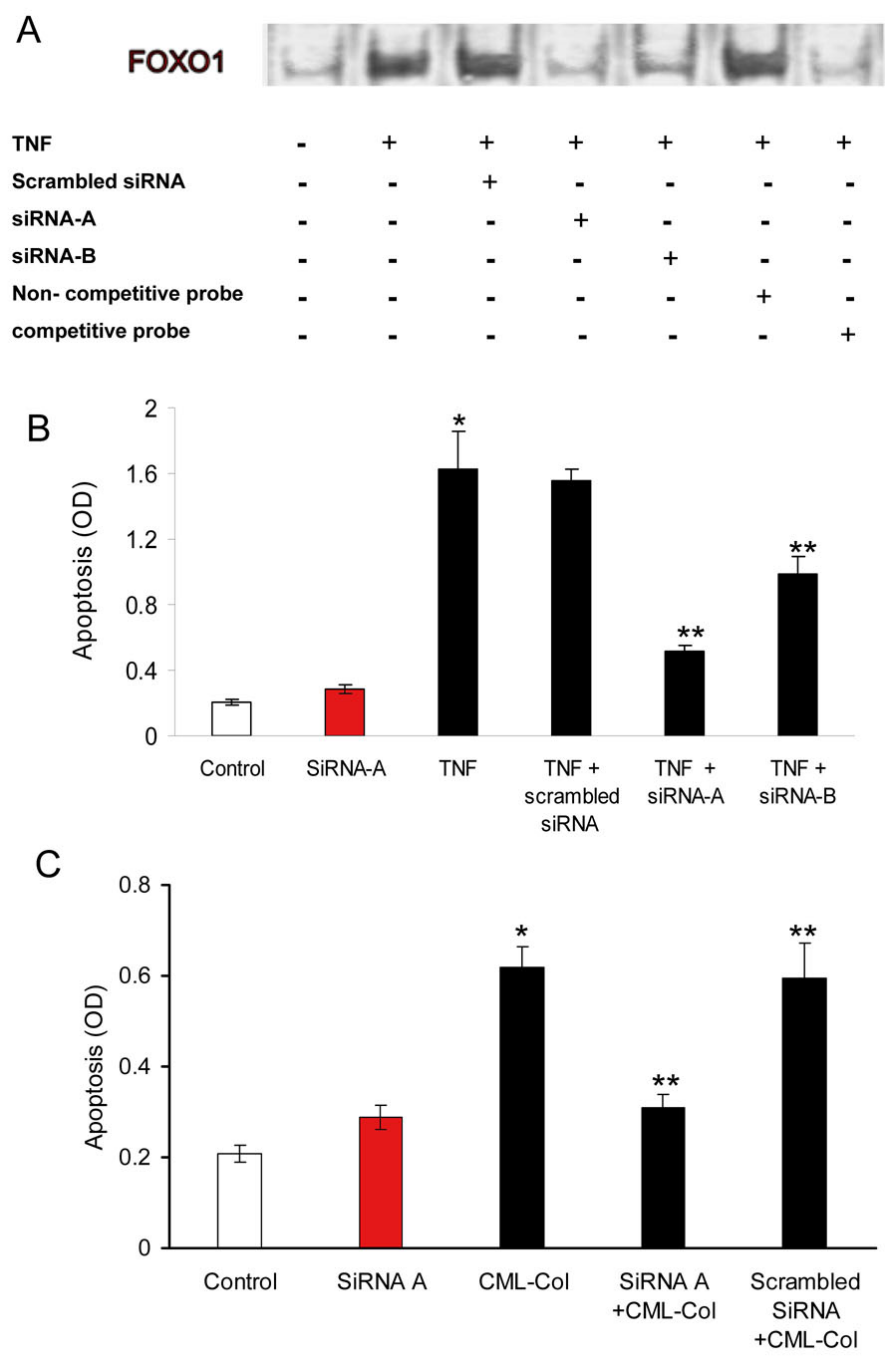
Forkhead box O1 (FOXO1) plays a major role in tumor necrosis factor-alpha (TNF-α)- or advanced glycation endproduct (AGE)-induced apoptosis in retinal pericytes. **A**: Primary cultures of retinal pericytes were transfected with different small interfering RNAs (siRNAs) for 48 h. Cells were then stimulated with TNF-α (20 ng/ml) for 1 h. After nuclear extraction, activation of FOXO1 was measured by electrophoretic mobility shift assay (EMSA). Unlabeled FOXO1 in excess was used as a competitive inhibitor. A probe with nonspecific sequence (nonspecific oligonucleotide) was used as a negative control. The experiment was performed three times with similar results. **B** and **C**: Primary cultures of retinal pericytes were transfected with different siRNAs for 48 h. Cells were then stimulated by TNF-α (20 ng/ml **B**) or carboxymethyllysine (CML)-collagen (200 μg/ml **C**) for 24 h. Apoptosis was determined by ELISA. Each value represents the mean of three replicates±standard error of the mean and is representative of three experiments. *, p<0.05 significantly differs from control; **, p<0.05, significantly differs from TNF-α- or CML-collagen-stimulated cells.

siRNA studies were next performed to establish the functional role of FOXO1 activation as an essential component in TNF-α-stimulated retinal pericyte apoptosis (Figure 3). Cells were pre-incubated for 48 h with siRNA, which was previously shown to be a strong FOXO1 silencer (siRNA-A), a partial silencer (siRNA-B), or scrambled siRNA, as discussed in the Methods section. In the presence of siRNA-A, binding activity of FOXO1 in response to TNF-α (20 ng/ml) stimulation decreased significantly, while siRNA-B had a partial effect and scrambled siRNA was not able to decrease FOXO1 DNA-binding activity (Figure 3A). To further investigate the functional role of FOXO1 activity on TNF-α- or CML-collagen-induced apoptosis, we measured pericyte apoptosis in the presence of different siRNAs. In the absence of TNF-α stimulation, there was little apoptosis, which was not affected by transfection with siRNA-A (Figure 3B). TNF-α induced an eightfold increase in the level of apoptosis (p<0.05). When transfected with strong silencing siRNA-A, apoptosis was reduced approximately 71% and was reduced by approximately 33% with moderately silencing siRNA-B (Figure 3A,B). CML-collagen stimulated a threefold increase in apoptosis (Figure 3C). Preincubation with FOXO1 siRNA-A reduced the level of apoptosis in response to CML-collagen by 60% (p<0.05). Transfection per se was not responsible for the reduced apoptosis since scrambled siRNA had no effect (Figure 3C).

**Figure 4:**
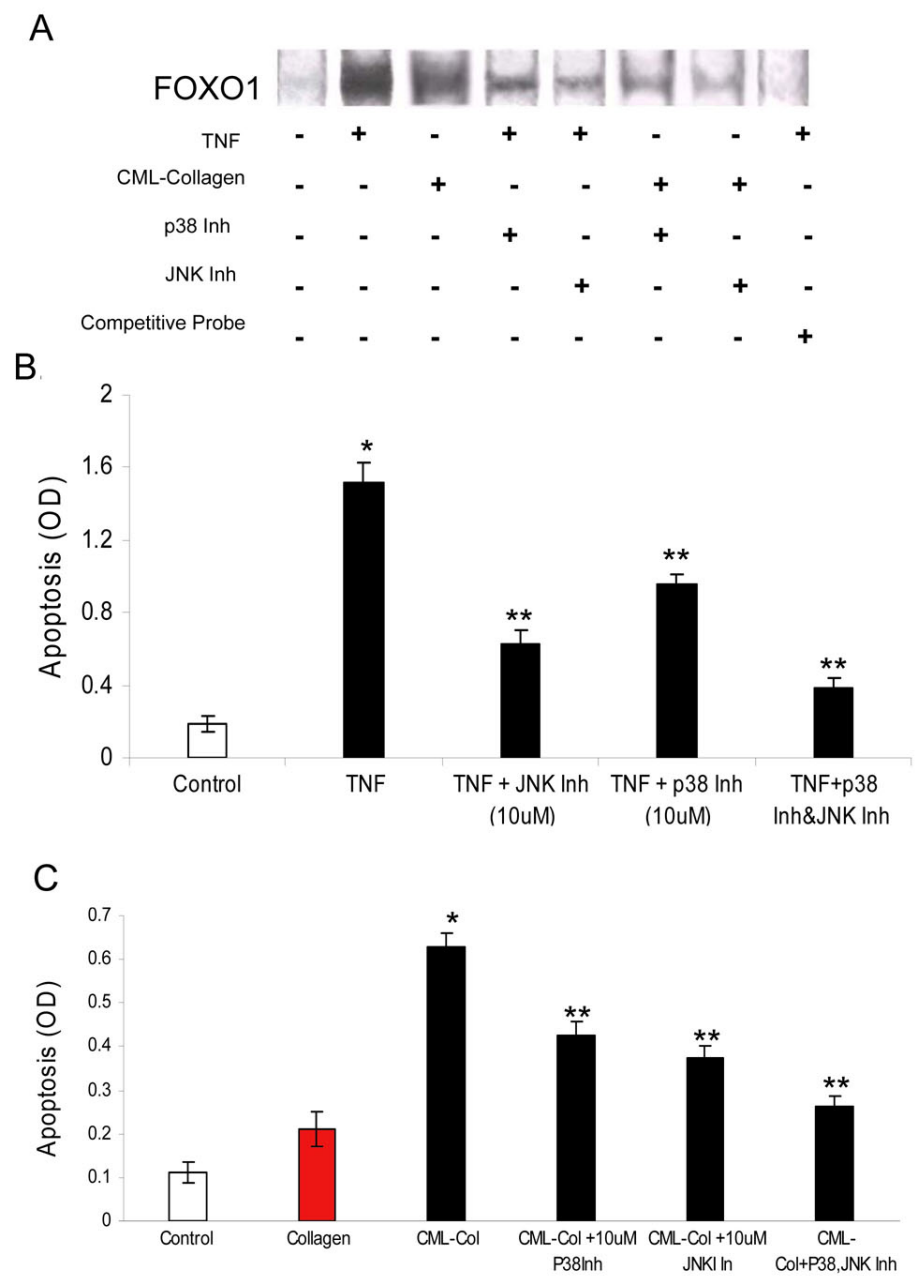
Jun N-terminal kinase (JNK) and P38 inhibitors block tumor necrosis factor (TNF)-induced Forkhead box O1 (FOXO1) activation and apoptosis. Primary bovine retinal pericytes were preincubated with or without the p38 inhibitor or JNK inhibitor for 2 h, followed by TNF-α (20 ng/ml) or carboxymethyllysine (CML)-collagen (200 μg/ml) stimulation. **A**: Activation of FOXO1 in response to TNF-α or CML-collagen stimulation (1 h) was measured by electrophoretic mobility shift assay (EMSA). Unlabeled FOXO1 in excess was used as a competitive inhibitor. **B**: Effect of JNK and P38 inhibitors on TNF-α-induced apoptosis (24 h) was determined by ELISA. **C**: Effect of JNK and P38 inhibitors on AGE-induced apoptosis (24 h) was determined by ELISA. Each value represents the mean of three replicates±standard error of the mean and is representative of three experiments. *, p<0.05 significantly differs from control (**B**) or control collagen (**C**); **, p<0.05, significantly differs from TNF-α- or CML-collagen-stimulated cells.

To gain insight into how TNF-α or an AGE might regulate FOXO1 activation and apoptosis, specific inhibitors of the MAP kinase signaling pathway were used. When retinal pericytes were preincubated with p38 or JNK inhibitors, TNF-α-induced FOXO1 DNA-binding activity was substantially reduced, as determined by EMSA (Figure 4A). A similar result was obtained in response to AGE-induced FOXO1 activation. Specificity was demonstrated by competitive inhibition with excess unlabeled probe (Figure 4A). The same inhibitors were then used to determine the functional role of p38 and JNK in TNF-α- or AGE-induced apoptosis (Figure 4B,C). TNF-α stimulated an eightfold increase in apoptosis. Apoptosis was reduced approximately 63% and 38% when cells were incubated with JNK and p38 inhibitors, respectively. However, when both inhibitors were used simultaneously, TNF-α-stimulated apoptosis was reduced by 77%, which was greater than either inhibitor alone (p<0.05; Figure 4B).

p38 inhibition decreased CML-collagen-induced apoptosis 34%, and the JNK inhibitor decreased apoptosis by 41%, both of which were statistically significant (p<0.05; Figure 4C). However, when both inhibitors were used simultaneously, CML-collagen-stimulated apoptosis was reduced by 58%, which was greater than either inhibitor alone (p<0.05; Figure 4C). Thus, both p38 and JNK arms of this signaling pathway are involved in CML-collagen-induced apoptosis. Doubling the concentration of p38 and JNK inhibitors did not significantly increase their inhibitory effect on TNF-α- or CML-collagen-stimulated apoptosis (data not shown).

**Figure 5:**
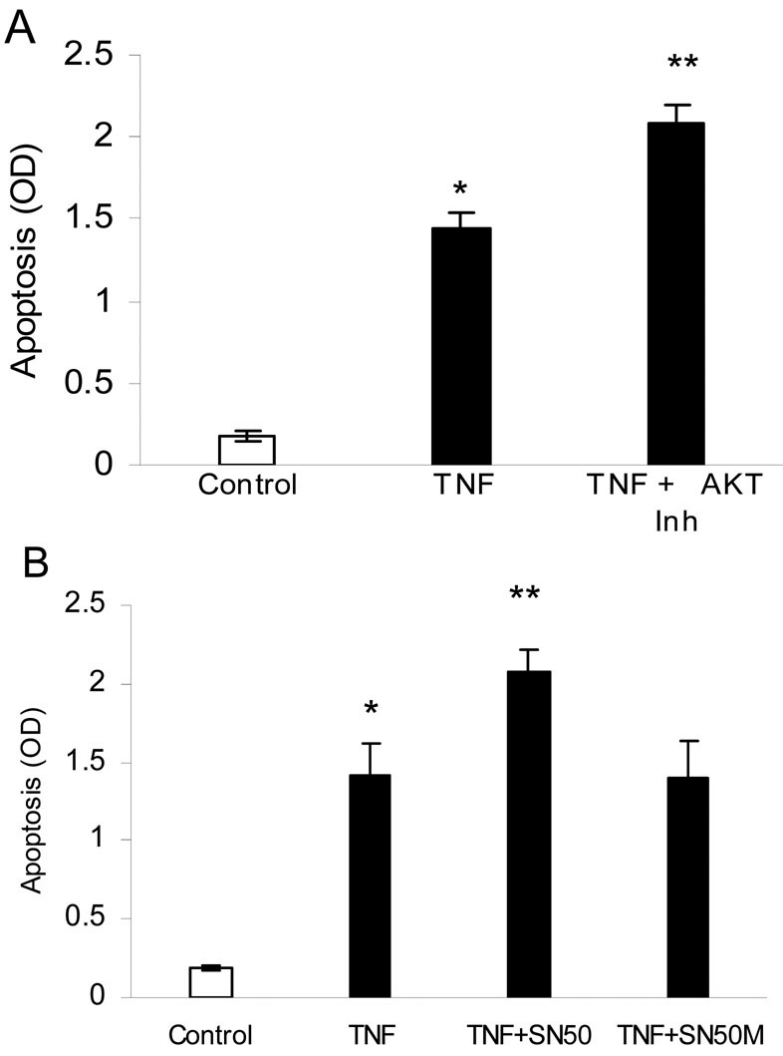
Inhibition of AKT or nuclear factor kappa B (NF-κB) enhances tumor necrosis factor-alpha (TNF-α)-induced apoptosis. Primary bovine retinal pericytes were preincubated with or without the Akt inhibitor, the specific NF-κB inhibitor (SN50), or the SN50M control peptide that lacks activity for 2 h, followed by TNF-α (20 ng/ml) stimulation in the presence of these molecules for 24 h. Apoptosis was determined by ELISA. **A**: Effect of Akt inhibitor on TNF-α-induced apoptosis was examined in pericytes. **B**: Effect of NF-κb inhibitor on TNF-α-stimulated apoptosis in pericytes. Each value represents the mean of three replicates±standard error of the mean and is representative of three experiments. *, p<0.05 significantly differs from the control; **, p<0.05, significantly differs from TNF-α-stimulated cells.

Akt, also known as protein kinase B, inhibits apoptosis through multiple mechanisms, one of which is the phosphorylation of FOXO1, which prevents FOXO1 translocation to the nucleus [19]. Similarly, NF-κB in many cell types is directly anti-apoptotic [34]. We further investigated apoptosis by determining whether Akt or NF-κB were affecting TNF-α-induced apoptosis. When Akt was inhibited, the capacity of TNF-α to stimulate apoptosis increased by 34% (Figure 5A), which was statistically significant (p<0.05). To investigate the role of NF-κB in apoptosis of pericytes, apoptosis of pericytes in the presence of TNF-α and NF-κB inhibitor was measured. Inhibition of NF-κB enhanced TNF-α-induced apoptosis by 41% (p<0.05; Figure 5B).

## Discussion

Retinal capillaries are composed of pericytes and endothelial cells. Pericytes and their processes located abluminally surround a single layer of endothelial cells located luminally. This unique localization allows pericytes to participate in the control of retinal vascular contractility and tone [35,36]. The studies of co-culture of endothelial cells and pericytes have suggested that pericytes have both anti-apoptotic and antiproliferative effects on endothelial cells [37]. The death of pericytes is an early event in the pathogenesis of diabetic retinopathy, and their loss is thought to compromise vessel architecture, resulting in nonperfused acellular capillaries and increased vascular leakiness [7]. The acellular capillaries, in turn, contribute to ischemia and hypoxia of the retinal tissue, a negative development in the pathogenesis of diabetic retinopathy [10]. Mechanisms proposed to account for pericyte apoptosis include rapid fluctuations of blood glucose [38], increased Bax expression during hyperglycemia [39], increased AGE formation [40], and TNF-α production [9].

Findings from this study suggest that FOXO1 is an important regulator of pericyte apoptosis that can work downstream of different apoptotic signals. Previously it was shown that activation of FOXO1 induces the global induction of pro-apoptotic genes and is thought to tilt the intracellular balance in an apoptotic direction [17,19,41]. In vitro experiments showed that in response to TNF-α and AGE stimulation retinal pericytes demonstrate high levels of FOXO1 DNA-binding activity. That this represents a mechanism for apoptosis in pericytes is supported by data that shows siRNA-mediated silencing of FOXO1 inhibits apoptosis. Interestingly, FOXO1 in diabetes is mostly known for its effect on insulin regulation of glucose production and insulin sensitivity [18,42,43]. Therefore, we suggest that the elevation of FOXO1 in addition to other effects can play a role in the pathogenesis of diabetic retinopathy through apoptosis of pericytes. This suggestion is supported by recently published findings that FOXO1 DNA-binding activity and nuclear translocation is enhanced in the microvascular cells in the retinas of type 1 and type 2 diabetic rats and that knockdown of FOXO1 by siRNA reduces formation of acellular capillaries and pericyte ghosts [21].

NF-κB activation in pericytes of diabetic retinas is closely linked to ocular complications of diabetes [44], and its activation has been suggested as one of the mechanisms for induction of apoptosis in retinal pericytes [44]. Our findings indicate that inhibition of NF-κB directly increases apoptosis in the pericytes in vitro, which suggests that NF-κB contributes to cell survival. However, NF-κB may also have indirect effects, particularly in a complex multicellular environment where the inflammatory effects of NF-κB may overcome its direct anti-apoptotic effects [34,45]. Recently we reported that apoptotic balance within a cell depends on the relative effects of NF-κB and FOXO1 activities [17]. TNF-α induces FOXO1, which then stimulates pro-apoptotic gene expression that occurs before apoptosis. Interestingly, TNF-α also activates NF-κB, a survival transcription factor. This would suggest that for TNF-α to induce apoptosis it must overcome the effect of NF-κB-regulated “cell survival” genes, a mechanism consistent with the need for FOXO1 activation. That NF-κB inhibitors enhanced TNF-α-stimulated apoptosis and FOXO1 siRNA inhibited TNF-α-stimulated apoptosis suggests that this paradigm exists in pericytes and may be particularly important in TNF-α-stimulated apoptosis. Similarly, Akt is known to block the functional activity of the FOXO transcription factors, including FOXO1 [42]. It has been shown that during retinal degeneration inactivation of Akt can contribute to apoptosis in photoreceptor cells [46]. This is in agreement with our findings that inhibition of Akt further enhanced TNF-α-induced apoptosis, which indicates that Akt functions as a survival factor in retinal pericytes. Meanwhile, inhibition of JNK and p38 MAP kinases substantially reduced the capacity of TNF-α to activate FOXO1. We also found that TNF-α-induced pericyte apoptosis was dependent upon these signaling pathways. Thus, JNK and p38 may have pro-apoptotic effects that include the activation of FOXO1.

Advanced glycation endproducts have been shown to stimulate cellular activity, including apoptosis through Receptor for Advanced Glycation Endproducts (RAGE) signaling, and to activate NF-κB [15,28]. NF-κB activation and apoptosis are stimulated in retinal endothelial cells by advanced glycation endproducts [47], and diabetic retinopathy increases NF-κB activation, which is associated with RAGE signaling in vivo [46]. Although it is well documented that NF-κB is directly anti-apoptotic in many cell types [45,48], NF-κB can indirectly promote apoptosis by inducing apoptotic factors [44]. We found that AGEs can stimulate activation of the transcription factor FOXO1, which is directly pro-apoptotic. Findings that inhibitors of JNK and p38 reduce AGE activation of FOXO1 suggests convergence with the TNF-α stimulation at the MAP kinase pathway or earlier. JNK and p38 may have pro-apoptotic effects that include the activation of FOXO1, which is consistent with our previous finding that showed that an AGE CML-collagen induces apoptosis of fibroblasts through RAGE signaling and activation of p38 and JNK MAP kinase pathways [22]. Thus, studies presented here indicate AGEs and TNF-α could contribute to diabetic retinopathy through FOXO1-mediated pericyte apoptosis.
